
JOURNAL INFORMATION
==============================
NLM Title Abbreviation: Tex Med J (Austin)
NLM Journal ID: 21130590R

Texas Medical Journal

ISSN: 0892-8495
Publisher: Biomedical Journal Digitization Project

ARTICLE INFORMATION
==============================
PMCID: PMC9640112
PMID: 36956547
Article version: 1
Subjects: Abstracts and Selections

Diet and Disease

Print publication date: 1910 Sep
Volume: 26
Issue: 3
First page: 109
Last page: 110
License: This work is free of known copyright restrictions. For more information, please see PMC Back Issue Digitization.
License URL: https://creativecommons.org/publicdomain/mark/1.0/

==============================

THE CHAS. H. PHILLIPS CHEMICAL CO.
NEW YORK and LONDON

PHILLIPS' MILK OF MAGNESIA
"THE PERFECT ANTACID"

SUPERIOR TO LIME WATER, BICARBONATE OF SODA, CHALK, ETC.
for correcting Hyperacid conditions—local or systemic.
Vehicle for Salicylates, Iodids, Balsams, etc.

TRADE MARK

PHILLIPS' REFINED CAMPHOR
PHILLIPS' WHEAT PHOSPHATES
PHILLIPS' DIGESTIBLE COCOA
PHILLIPS' PHOSPHO-MURIATE OF QUININE
PHILLIPS' SYRUP OF WHEAT PHOSPHATES
PHILLIPS' EMULSION OF COD LIVER OIL

has been created and will meet annually at the Institute. The following have accepted the invitation to serve as members of this body: Dr. Samuel G. Dixon, Harrisburg, Pa.; Dr. S. McC. Lindsay, New York City; Dr. William H. Baldwin, Washington, D. C.; Dr. Hermann M. Biggs, New York City; Dr. William H. Welch, Baltimore, Md.; Dr. Theobald Smith, Boston, Mass.; Dr. Gideon Wells, Chicago, Ill.; Dr. Simon Flexner, New York City; Dr. James A. Miller, New York City; Dr. Lawrence Brown, Saranac, N. Y.; Dr. Henry Baird Favell, Chicago, Ill., and Dr. James Pratt, Boston, Mass.

### Diet and Disease.

Dr. Robert Bell, in a recent lecture to the Psycho-Therapeutic Society, at the Baxton Hall, Westminister, said that man, by his absurd diet, courted disease. He loaded his stomach with food that had been deprived of most of its nutritive value. To obtain sufficient nourishment he had to eat too much, and a great proportion of the ingested matter underwent rapid decomposition of the most noxious kind, giving rise to virulent toxins which exerted a most pernicious influence on health. When he commenced his medical career, cancer was not nearly so prevalent as today. Then, however, there was not nearly so much flesh meat eaten as now. Products of the vegetable kingdom, together with milk, eggs, and cheese, were ideal foods. Fruits and seeds contained radio-active elements when uncooked. These foods when ripe—that is, sufficiently cooked by the sun—should constitute our staple diet. When cooked, their vital and vivifying constituent was nullified. He had known more than one instance of habitual drunkenness completely cured by abstinence from flesh food.—*British Health Review.*

---

### Publisher's Department.

---

TO ELEVATE VITAL RESISTANCE.—In the incipient or so-called pre-tubercular stages of tuberculosis, when the most significant symptom is a faltering digestive process, soon reflecting itself in a characteristic nutritional decline, Seng often has a unique value, for it spurs the digestive organs in a very substantial way that almost invariably insures an increased food appropriation and a consequent highly desirable elevation of vital resistance.

---

AN IDEAL OINTMENT BASE.—As an ointment base with which to incorporate any active drug or preparation, no more desirable compound can be found than Unguentine, since it is non-irritating and never becomes rancid and, at the same time, possesses sufficient astringent qualities to facilitate rapid healing.

---

BATTLE & Co., of St. Louis, have just issued No. 13 of their series of charts on dislocations. This series forms a most valuable and interesting addition to any physician's library. They will be sent free of charge on application, and back numbers will also be supplied. If you have missed any of these numbers, better write Battle & Co. for them before the supply is exhausted.

---

FOR YEARS I have used Tongaline and I consider it a valuable arrow in the quiver with which to fight that hydra-headed evil, rheumatism, especially when there are any gouty complications.

---

I HAVE PERSONALLY derived great benefit from the use of Tongaline for rheumatism. Among the many advantages of Tongaline over the salicylates is its palatability and the fact that it is not trying on the stomach, hence, wherever the salicylates are indicated, I invariably prescribe Tongaline.
